# Disparities in E-Cigarette and Tobacco Use Among Adolescents With Disabilities

**DOI:** 10.5888/pcd17.200161

**Published:** 2020-10-29

**Authors:** Angela Senders, Willi Horner-Johnson

**Affiliations:** 1 Oregon Health and Science University, Oregon Office on Disability and Health, Portland, Oregon; 2 OHSU-PSU School of Public Health, Portland, Oregon; 3 National University of Natural Medicine, Helfgott Research Institute, Portland, Oregon

## Abstract

**Introduction:**

In 2019, nearly 30% of US high-school students reported current (past 30 day) e-cigarette use. Adolescents with disabilities are consistently more likely to smoke cigarettes compared with their nondisabled peers, yet little is known about their use of other forms of tobacco, including e-cigarettes. We compared the prevalence of tobacco use (e-cigarettes, cigarettes, little cigars, large cigars, hookahs, and smokeless tobacco) among high school students with at least 1 disability to those without disability.

**Methods:**

Data were from the 2015 and 2017 Oregon Healthy Teens survey, a statewide representative sample of 11th-grade students. We estimated the prevalence of current (past 30 day) tobacco use by product type and disability status (yes or no). We used multivariable Poisson regression to estimate prevalence ratios measuring the association between disability status and current tobacco use, by product: 1) combustible products only, 2) e-cigarettes only, and 3) dual use of combustibles and e-cigarettes.

**Results:**

Students with disabilities were more likely to use a variety of tobacco products compared with their nondisabled peers, including cigarettes (12.3% vs 5.4%), little cigars (7.0% vs 5.4%), hookahs (6.2% vs 3.8%), and e-cigarettes (18.3% vs 12.3%). In adjusted models, students with a disability were more likely to report using combustibles only (adjusted prevalence ratio [aPR], 1.55; 95% CI, 1.31–1.84), e-cigarettes only (aPR, 1.36; 95% CI, 1.16–1.59), or dual use (aPR, 1.52; 95% CI, 1.29–1.80) compared with nondisabled students.

**Conclusion:**

Effective tobacco control programs should target populations with the greatest burden of tobacco use. Results suggest that tobacco prevention and reduction efforts should explicitly include adolescents with disabilities and employ accommodations that support their participation in program activities.

**Summary**

**What is already known on this topic?**

In 2019, nearly 30% of US high-school students reported current (past 30 day) e-cigarette use. Equitable tobacco control programs should target populations with the greatest burden of exposure, yet we are unaware of any studies of e-cigarette use among adolescents with disabilities.

**What is added by this report?**

We observed disparities in tobacco use between adolescents with and without disabilities for a variety of tobacco products, including e-cigarettes.

**What are the implications for public health practice?**

Tobacco prevention and control efforts that do not target high-risk populations can exacerbate existing disparities. Our findings suggest that prevention efforts should include adolescents with disabilities.

## Introduction

Adolescent cigarette smoking has declined in recent years, only to be offset by an increase in the use of e-cigarettes and vaping devices (hereinafter referred to as electronic nicotine delivery systems, or ENDS) (1,2). In 2019, 27.5% of high school students reported current (past 30 day) use of ENDS, up from 11.7% in 2017 (2). This surge was partially fueled by the perception among many adolescents that ENDS are fashionable, fun, and safe (3). Although the understanding of the long-term health effects of ENDS use continue to develop, some clear harms have been documented. Specifically, nicotine can have powerful and lasting effects on the developing brain, and adolescent exposure is associated with impaired cognition, attention, memory, and mood (4). Adolescents are also susceptible to a strong rewarding effect of nicotine; young smokers are more likely to become addicted than adults, and adolescent exposure to nicotine is associated with subsequent use of other addictive substances (4). In addition to nicotine, the aerosol from ENDS can contain chemical irritants and carcinogens, including heavy metals, formaldehyde, acetone, ultrafine particulate matter, and polycyclic aromatic hydrocarbons (4). Use of ENDS has resulted in severe lung damage and in some cases death (5), and the US Surgeon General has declared ENDS use among young people a major public health concern (4).

To effectively reduce the use of combustible tobacco products and ENDS, prevention and control efforts should target population groups that bear the greatest burden of tobacco use (6). Adolescents who report having a disability are consistently more likely to smoke cigarettes compared with their nondisabled peers (7), and this disparity continues into adulthood (8). The published evidence of disability-related disparities in tobacco use is primarily limited to cigarettes. Little is known about the full spectrum of tobacco product use among adolescents with disabilities. In particular, we are unaware of any studies that have estimated ENDS use among adolescents in this vulnerable population.

As a population, people with disabilities are subject to health disparities, and efforts to better understand from where preventable health inequalities stem are essential (9). Therefore, we examined the use of cigarettes, cigars, hookahs, smokeless tobacco, and ENDS in 2015 and 2017 in a statewide representative sample of Oregon 11th-grade students. We compared the age of initiation and the prevalence of current tobacco use in students with self-reported disabilities to those who did not report disabilities.

## Methods

### Data source and analytic sample

The state of Oregon monitors the health and well-being of adolescents with an anonymous biennial survey. The Oregon Healthy Teens survey is administered to 8th- and 11th-grade public school students to collect data on a variety of topics, including behaviors related to diet, exercise, sexual activity, alcohol consumption, and smoking. Data are weighted to account for the multistage sampling design and to represent the statewide population of students in each grade.

We conducted a cross-sectional analysis of pooled data collected from 11th-grade students in 2015 and 2017. We did not include 8th graders because they were not asked about disability status. Across the 2 years 25,503 11th-graders from 169 schools in 35 (of 36) Oregon counties participated in the survey. Students were excluded from our analyses if they were missing data for disability status (n = 1,154; 4.5%), tobacco or ENDS use (n = 1,877; 7.4%), or other independent variables of interest (n = 2,244; 8.8%). The remaining 20,228 students were included in the analytic study sample (Figure).

**Figure:**
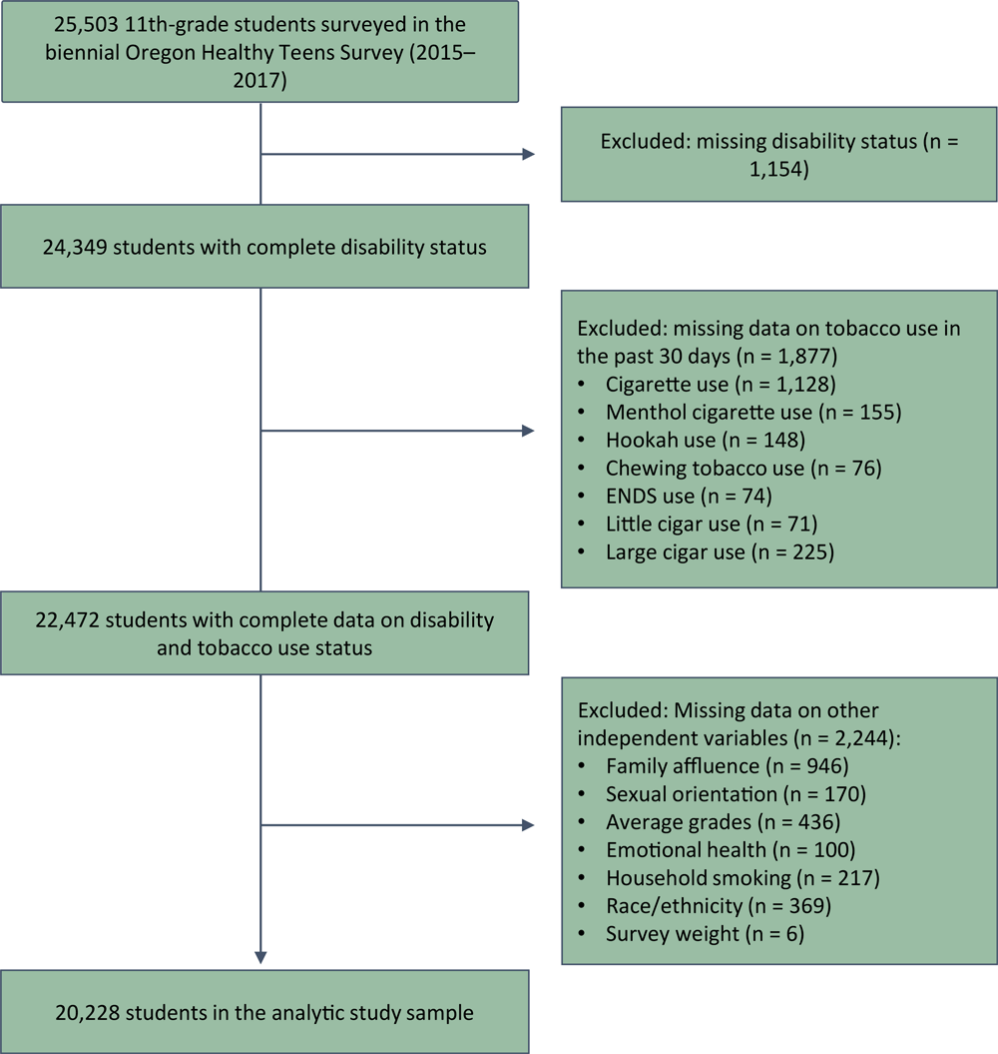
Logic model showing determination of analytic study sample of 11th-grade students, Oregon Healthy Teens survey, 2015 and 2017. Abbreviation: ENDS, electronic nicotine delivery systems.

### Dependent variable: tobacco use

Students were queried on the use of cigarettes, menthol cigarettes, little cigars, large cigars, chewing tobacco/snuff/dip, hookah/water pipes, and e-cigarettes or other vaping products (ie, ENDS) within the past 30 days. Additionally, we created 3 mutually exclusive dichotomous dependent variables reflecting categories of current tobacco use: 1) combustible products only, 2) ENDS only, and 3) dual use of combustibles and ENDS. Combustible products included cigarettes, menthols, hookahs, and little and large cigars. For all variables, current use was defined as any use within the 30 days before the survey (yes or no).

Students were also asked the age at which they smoked their first cigarette and the age at which they first used any other tobacco product (including ENDS). Possible responses included: never, 8 years or younger, 9 to 16 years by 1-year increments, or 17 years or older. For both questions, we dichotomized responses among ever users as less than or greater than or equal to the median age at first use, which was 14 years.

### Independent variables

Disability status was assessed with 6 questions established by the US Department of Health and Human Services as the minimum standard for disability data collection in health surveys (10). These binary questions ask whether a student has a visual, hearing, cognitive, mobility, self-care, and/or independent living disability. Because sample sizes for individual disabilities were small and 30% of students with a disability reported having more than 1 disability, we defined our independent variable as student report of at least 1 disability (yes or no).

We controlled for known sociodemographic risk factors of tobacco use that may confound the associations of interest, including race/ethnicity (non-Hispanic White, Hispanic, and other race non-Hispanic), gender identity (female, male, nonbinary identity), sexual orientation (straight, sexual minority), family affluence (low, middle, high), and whether or not a member of the adolescent’s household smokes (no one else smokes, someone smokes but not inside the house, someone smokes inside the house) (11). Race/ethnicity was considered a marker of social stressors associated with smoking risk; categories were combined because of small sample sizes. Similarly, nonbinary gender identities were only queried in 2017 and categories were combined to manage small cell sizes. Family affluence was assessed with the Family Affluence Scale II (12), a measure designed for use among adolescents.

Additional factors may lie on the causal pathway between disability status and tobacco use, so we reported a third model that adjusted for self-reported mental health (good to excellent or fair to poor), school performance (grades of mostly As and Bs; yes or no), and stressful life events. Students who experienced any of the following in the 12 months before the survey were categorized as having had a stressful life event (yes or no): feeling unsafe to go to school, threatened with a weapon, fighting at school, bullied at school, forced or pressured to have sex, sexual contact with adult, and hit by an adult or partner.

### Statistical and sensitivity analyses

Unweighted counts, weighted proportions and 95CIs for sociodemographic characteristics and tobacco use were calculated for the overall analytic sample and by disability status. We used design-based F-tests to assess bivariate associations between sociodemographic or tobacco variables and disability status. Poisson regression with robust variance was used to estimate adjusted prevalence ratios (aPRs) and 95% CIs as the measure of association between current (past 30 day) tobacco use and disability. For each dependent variable we built 3 models: 1) unadjusted, 2) adjusted for theoretical confounders, and 3) further adjusted for potential mediators. A *P* value of .05 or less was considered significant. All analyses were conducted in Stata version 15.1 (StataCorp LLC).

The most prevalent type of disability reported was cognitive. To assess whether the association between disability status and tobacco use was driven primarily by this group, we excluded students who reported cognitive as their only disability (n = 2,769). We then recalculated the unadjusted prevalence of tobacco use by product and reran final regression models.

## Results

Most students identified as white (62.4%) and straight (85.7%) and reported middle or high family affluence (90.5%), good to excellent mental health (70.2%), and grades of mostly As and Bs (71.3%) (Table 1). The prevalence of self-reported disability was 28.6%. The most prevalent type of disability was cognitive (n = 4,485; 22.7%), followed by independent living (n = 1,625; 8.1%), vision (n = 942; 4.7%), mobility (n = 476; 2.5%), hearing (n = 396; 2.1%), and personal care (n = 155; 0.7%). When comparing students with at least 1 disability to those without, we found significant differences across all sociodemographic characteristics. In particular, students with disabilities were more likely to report sexual minority status, fair to poor mental health, poor school performance, and the experience of stressful life events.

**Table 1:** Sociodemographic Characteristics of a Statewide Representative Sample of 11th-Grade Students, by Disability Status, Oregon Healthy Teens Survey, 2015 and 2017^a^

Characteristic	Total (N = 20,228)		Disability (n = 5,784)		No Disability (n = 14,444)		*P* Value^b^
**Mean age, (SD), y**	NA	16.6 (1.1)	NA	16.6 (1.2)	NA	16.6 (1.0)	NA
**Race/ethnicity**							
White, Non-Hispanic	12,762	62.4 (58.4–66.3)	3,435	58.0 (53.4–62.4)	9,327	64.3 (60.1–68.2)	<.001
Hispanic	4,517	23.2 (19.7–27.2)	1,439	26.7 (22.4–31.5)	3,078	21.8 (18.4–25.7)	
Other race, non-Hispanic	2,949	14.3 (12.6–16.3)	910	15.3 (13.2–17.6)	2,039	13.9 (12.0–16.0)	
**Gender identity**							
Female	10,240	50.7 (49.4–51.9)	3,497	60.9 (58.5–63.2)	6,743	46.5 (45.0–48.0)	<.001
Male	9,433	46.4 (45.2–47.6)	2,004	33.9 (31.6–36.3)	7,429	51.5 (50.0–53.0)	
Other^c^	555	2.9 (2.4–3.6)	283	5.2 (4.3–6.4)	272	2.0 (1.6–2.5)	
**Sexual orientation**							
Straight	17,398	85.7 (84.5–86.7)	4,283	73.0 (70.7–75.1)	13,115	90.8 (89.9–91.7)	<.001
Sexual minority	2,830	14.3 (13.3–15.5)	1,501	27.0 (24.9–29.3)	1,329	9.2 (8.3–10.1)	
**Family affluence^d^**							
Low	1,912	9.5 (8.4–10.8)	738	12.7 (11.2–14.2)	1,174	8.2 (7.1–9.5)	<.001
Middle	7,186	35.9 (34.2–37.6)	2,275	39.4 (37.5–41.3)	4,911	34.4 (32.5–36.4)	
High	11,130	54.6 (52.0–57.2)	2,771	48.0 (45.4–50.6)	8,359	57.4 (54.5–60.2)	
**Smoker in household^e^**							
No	14,420	71.2 (69.2–73.2)	3,628	63.0 (60.4–65.6)	10,792	74.5 (72.5–76.4)	<.001
Yes, not inside	4,814	23.8 (22.1–25.6)	1,706	29.5 (27.3–31.8)	3,108	21.5 (19.9–23.2)	
Yes, smokes inside	994	5.0 (4.4–5.6)	450	7.5 (6.4–8.8)	544	4.0 (3.5–4.5)	
**Mostly A and B grades**	14,319	71.3 (68.7–73.8)	3,356	58.6 (56.1–61.0)	10,963	76.5 (73.9–79.0)	<.001
**Stressful events^f^**	9,024	44.5 (42.9–46.2)	3,744	64.6 (62.8–66.4)	5,280	36.3 (34.3–38.3)	<.001
**Mental health**							
Good or excellent	14,375	70.2 (68.5–71.8)	2,416	40.7 (38.4–43.0)	11,959	82.2 (80.8–83.5)	<.001
Fair or poor	5,853	29.8 (28.2–31.5)	3,368	59.3 (57.0–61.6)	2,485	17.8 (16.5–19.2)	

Abbreviation: NA, not applicable.

a Values are weighted percentages and 95% CIs based on a representative sample of 11th-grade students in Oregon, unless otherwise indicated.

b *P* value for F-test of weighted percentages.

c Nonbinary identifiers were only surveyed in 2017.

d Determined by using the Family Affluence Scale II (12).

e Includes vaping by household member in 2017 only.

f Stressful events include the report of any of the following: feeling unsafe to go to school, threatened with a weapon, fighting at school, bullied at school, forced or pressured to have sex, sexual contact with an adult, or hit by adult or partner.

Overall, 19.2% of 11th grade students had used a tobacco product in the 30 days before the survey; 7.4% reported smoking cigarettes, and 14.1% reported using ENDS (Table 2). The current use of other tobacco products ranged from 2.2% (large cigars) to 5.9% (little cigars). When assessed by disability status, the estimated proportion of cigarette use among students with disability was more than double that of students without (12.3% vs 5.4%, respectively). We found a similar pattern for use of little cigars (7.0% disability vs 5.4% no disability), hookah (6.2% disability vs 3.8% no disability) and ENDS (18.3% disability vs 12.3% no disability). Students with disability also reported a higher prevalence of dual use of a combustible product and an electronic delivery system within the 30 days before survey (11.0% disability vs 6.6% no disability). We observed no significant differences in smokeless tobacco or large cigar use by disability status.

**Table 2:** Current (Past 30-Day) Use of Tobacco or Nicotine Products in a Statewide Representative Sample of 11th-Grade Students, Oregon Healthy Teens Survey, 2015 and 2017

Type of Use	Total (N = 20,228)		Disability (n = 5,784)		No Disability (n = 14,444)		*P* Value^b^
	N	% (95% CI)^a^	N	% (95% CI)^a^	N	% (95% CI)^a^	
**Use of individual products^c^**							
Cigarettes^d^	1,510	7.4 (6.5–8.5)	744	12.3 (10.9–13.9)	766	5.4 (4.5–6.6)	<.001
Little cigars	1,203	5.9 (5.3–6.6)	448	7.0 (6.1–8.1)	755	5.4 (4.8–6.1)	.001
Large cigars	455	2.2 (1.9–2.6)	164	2.6 (2.1–3.2)	291	2.1 (1.7–2.5)	.10
Hookah	946	4.5 (4.0–5.1)	384	6.2 (5.3–7.3)	562	3.8 (3.3–4.4)	<.001
Smokeless tobacco	819	3.7 (3.2–4.3)	251	4.1 (3.2–5.1)	568	3.6 (3.1–4.2)	.31
ENDS^e^	2,855	14.1 (12.9–15.3)	1,098	18.3 (16.6–20.1)	1,757	12.3 (11.1–13.7)	<.001
**Usage categories^f^**							
Any product^g^	3,959	19.2 (17.9–20.6)	1,509	25.1 (23.1–27.2)	2,450	16.8 (15.4–18.2)	<.001
Combustibles only	963	4.6 (4.1–5.1)	390	6.5 (5.6–7.7)	573	3.8 (3.4–4.2)	<.001
ENDS only	1,278	6.2 (5.6–6.9)	433	7.3 (6.4–8.4)	845	5.7 (5.1–6.4)	.001
Combustibles and ENDS	1,577	7.9 (7.0–8.8)	665	11 (9.7–12.4)	912	6.6 (5.7–7.7)	<.001

Abbreviations: ENDS, electronic nicotine delivery systems.

a Weighted percentages and 95% CIs based on a representative sample of 11th-grade students in Oregon.

b *P* value for *F* test of weighted percentages.

c Categories are not mutually exclusive; 9.6% of the total sample used 2 or more products.

d Includes menthols.

e Includes e-cigarettes and other vaping devices.

f Usage categories are mutually exclusive.

g Includes cigarettes and menthols, little and large cigars, hookah, smokeless tobacco, or ENDS.

Among cigarette-smoking students in this sample, 38.3% of students with a disability reported smoking their first cigarette before the age of 14, compared with 33.4% of students without disability (*P* = .008). Similarly, 20.7% of students with a disability initiated the use of a tobacco product other than cigarettes (including ENDS) before turning 14, compared with 14.6% of students without disability (*P* < .001).

In unadjusted Poisson regression models, disability status was significantly associated with the current use of 1) combustible tobacco products only, 2) ENDS only, and 3) dual combustible and ENDS use (Table 3). After controlling for sociodemographic factors, the prevalence ratios for the models of combustibles only and dual use only were attenuated yet remained significant. The adjusted prevalence of combustible only use was 55% higher among students with disability compared with students without (aPR 1.55; 95% CI, 1.31–1.84), and the prevalence of dual use of combustibles and ENDS was 52% higher (aPR 1.52; 95% CI, 1.29–1.80). After adjusting for potential mediating factors of mental health, school performance, and stressful life events, the point estimates for these 2 outcomes were further attenuated; the prevalence ratio for combustible only use was reduced from 1.55 to 1.28 (95% CI, 1.05–1.56), and the prevalence ratio for dual use was reduced from 1.52 to 1.04 (95% CI, 0.88–1.24), the latter being no longer significant.

**Table 3:** Association Between Current (Past 30-Day) Tobacco or Nicotine Use and Disability Status in a Statewide Representative Sample of 11th-Grade Students (N = 20,228), Oregon Healthy Teens Survey, 2015 and 2017^a^

Variable	Combustible Use Only^b^	ENDS Use Only^c^	Dual Use of Combustibles and ENDS
	Multivariable Prevalence Ratio (95% CI)		
**Disability**			
No	1 [Reference]		
Yes	1.55 (1.31–1.84)	1.36 (1.16–1.59)	1.52 (1.29–1.80)
**Race/ethnicity**			
White, Non-Hispanic	1 [Reference]		
Hispanic	0.97 (0.79–1.20)	0.78 (0.63–0.96)	0.76 (0.61–0.96)
Other race, non-Hispanic	0.95 (0.71–1.28)	0.92 (0.71–1.19)	0.81 (0.62–1.06)
**Gender identity**			
Female	1 [Reference]		
Male	1.16 (0.96–1.40)	1.36 (1.16–1.60)	1.33 (1.15–1.56)
Other^d^	1.79 (0.89–3.59)	1.03 (0.66–1.62)	0.71 (0.45–1.11)
**Sexual orientation**			
Straight	1 [Reference]		
Sexual minority	1.1 (0.83–1.46)	0.77 (0.60–1.00)	1.34 (1.12–1.61)
**Family affluence** e			
High	1 [Reference]		
Middle	1.25 (1.02–1.54)	0.88 (0.75–1.03)	1.15 (0.93–1.43)
Low	1.39 (1.09–1.78)	0.71 (0.52–0.98)	1.58 (1.16–2.16)
**Household smoking^f^**			
No one else smokes	1 [Reference]		
Someone smokes, not inside	1.85 (1.49–2.30)	1.39 (1.17–1.65)	1.89 (1.63–2.19)
Someone smokes inside	1.55 (1.14–2.11)	2.47 (1.90–3.22)	1.80 (1.40–2.32)

Abbreviation: ENDS, electronic nicotine delivery systems.

a Crude rates of current use of combustibles only was 1.73 (95% CI, 1.46–2.07), of ENDS only was 1.28 (95% CI, 1.10–1.48), and of both combustibles and ENDS was 1.66 (95% CI, 1.43–1.93).

b Includes cigarettes and menthols, little and large cigars, and hookahs/water pipes.

c Includes e-cigarettes and vaping products.

d Nonbinary identifiers were only surveyed in 2017.

e Determined by using the Family Affluence Scale II (12).

f Includes vaping by household member in 2017 only.

In contrast to the combustibles only and the dual use models, the crude association between disability status and ENDS only use was not as strong (PR, 1.28; 95% CI, 1.10–1.48). The prevalence ratio for the ENDS only model increased slightly after controlling for potential confounders (aPR, 1.36; 95% CI, 1.16–1.59). After adjusting for possible mediating factors, the association between disability and ENDS only use was attenuated and no longer significant (aPR 1.13; 95% CI, 0.95–1.36).

After excluding students whose only disability was cognitive, significant differences in tobacco product use between students with and without disabilities were materially unchanged. Likewise, the prevalence ratios for combustible use only, ENDS use only, and dual use of combustibles and ENDS were similar to findings of the primary analysis.

## Discussion

The deleterious health consequences of tobacco use are extensive and well documented (13), and those of ENDS continue to unfold (14). Most regular smokers (88%) begin before age 18 (11), and considerable work has been conducted to understand patterns of use among adolescents. Socially disadvantaged adolescents bear a disproportionate burden of tobacco use, including those who perform poorly in school, identify as a sexual minority, are of lower socioeconomic status, or whose parental figures demonstrate a lack of support or involvement (11). Our investigation adds to this literature by assessing the prevalence of tobacco and ENDS use among another vulnerable subpopulation, adolescents with disabilities. To our knowledge, our study is the first to describe tobacco use by product type among students with disabilities. We found that students with disabilities were more likely to use cigarettes, little cigars, hookahs, and ENDS compared with their peers without disabilities. Even after adjusting for potential confounding factors, students with a disability were more likely to report being a current user of combustible products (only), ENDS (only), or both, compared with nondisabled students. Students with disabilities also reported initiating tobacco use at an earlier age than students without disabilities.

In 2017, an estimated 19.6% of US high school students currently used (past 30 days) any tobacco product (1). Consistent with these findings, we estimated that from 2015 to 2017, 19.2% of Oregon 11th graders currently used any tobacco product. However, that overall prevalence estimate masked tobacco use disparities between students with a disability and those without; 25% of Oregon 11th-graders with a disability used a tobacco product in the previous 30 days compared with 16.8% of students without disability. This pattern persisted for most individual tobacco products. These results align with prior population-based estimates of tobacco use in students with and without disabilities (15–20), 2 of which were conducted in US samples (19,20). Blum et al used cross-sectional data from the 1994–1995 wave of the National Longitudinal Study of Adolescent Health to assess the association between disability status and regular smoking (at least 1 cigarette per day in the previous 30 days) (19). They reported that 7th- through 12th-graders with an emotional, mobility, or learning disability were significantly more likely to report regular cigarette smoking compared with nondisabled students. Jones and Lollar analyzed data from the Centers for Disease Control and Prevention’s (CDC’s) 2005 Youth Risk Behavior Survey and found the odds of current cigarette smoking (past 30 days) were 50% higher among 9th- through 12th-graders with a disability or chronic health problem compared with students without such health concerns. Comparing our results to these and other international findings is difficult because of substantial variability in how disability and tobacco use are defined across studies, as well as cultural differences in tobacco use between countries. Despite these challenges, population-based surveys consistently demonstrate that the prevalence of tobacco use is higher among adolescents with disability compared with their peers (7). Our work expands on these findings by disaggregating tobacco use by product type and providing estimates of ENDS use by disability status among 11th-grade students.

Some of the excess burden we observed among students with a disability may be explained by differences in factors that influence tobacco use among adolescents. We found that students with at least 1 disability were substantially more likely to experience stressful life events, including violence, abuse, peer pressure, or bullying. They were also more likely to report fair to poor mental health status and poorer performance in school. We conceptualized these factors as potential mediators because they likely lie on the causal pathway between disability and tobacco use. Adolescents with disabilities often face stigma, discrimination, exclusion, and other forms of social adversity (21). They may also face challenges in having their academic needs met, resulting in lower grades. These stressors are not benign and may contribute to poor mental health among adolescents with disabilities. We did not conduct a formal mediation analysis to test these hypotheses, because longitudinal data would better serve this purpose. However, when adjusting for these factors (Model 3), the magnitude of the association between disability and tobacco use was attenuated, and for users of ENDS only and dual users of ENDS and combustibles the association was no longer significant. These findings suggest that efforts to improve the high school experience for students with disabilities could potentially result in lower uptake of tobacco products in the first place. Further research is needed to explore this hypothesis.

Our study has limitations. Our data are cross-sectional, so causality between disability and tobacco use cannot be evaluated. Surveys were administered only in public schools, limiting the generalizability of our findings to students enrolled in these institutions. Data were self-reported and could be subject to recall or reporting bias. Students may have been hesitant to disclose their tobacco use behavior, although we expect that the anonymity of the survey minimized this risk and that any reluctance to report tobacco use was nondifferential with respect to disability status. We did not conduct a formal mediation analysis; future data collection efforts should be designed with such analyses in mind. The 6 disability questions established by the US Department of Health and Human Services have not been cognitively tested in adolescent populations, and we do not know how adolescents interpret them. Indeed, the prevalence of any disability and of cognitive disabilities in our data set is higher than that of American adults aged 18–44 (22). This finding suggests that some of the disability data captured by the Oregon Healthy Teens survey may be misclassified or transient. For example, a portion of cognitive disabilities may reflect challenges related to the adolescent experience that do not apply later in life. Future validation of these questions among adolescents is essential. Finally, people with disabilities are a heterogeneous population, yet small sample sizes prevented us from disaggregating disability by type. Our sensitivity analyses suggest that observed associations were not solely a result of the most prevalent disability type in our sample; nevertheless, larger samples are needed to better understand differences in smoking behaviors across disability types and to tailor interventions appropriately. 

Despite these limitations, we were able to estimate the prevalence of tobacco use in a marginalized population — students with disabilities — and identify disparities that have implications for tobacco prevention and control efforts. Oregon is one of the few states to include any disability questions in a statewide adolescent health survey, and the only one to our knowledge to have added the 6 standard disability identifiers.

### Public health implications

Tobacco use is the foremost cause of preventable illness and death in the United States (13). Marginalized populations, including people with disabilities, bear a disproportionate burden of tobacco-related health consequences. Adults with disabilities are more likely to smoke and to have tobacco-related illnesses that can exacerbate disability, decrease quality of life, and shorten the lifespan compared with adults without disabilities (9). Most regular tobacco users begin in adolescence, and tobacco use disparities by disability status are present even at these younger ages. Thus, preventing tobacco use initiation among adolescents with disabilities should be a major public health aim.

Tobacco prevention and control efforts that do not target high-risk populations can exacerbate existing disparities (6). For example, public health campaigns targeting smokers as a population have contributed to a decreased prevalence of smoking among educated and higher-income adults, with little change among those of lower socioeconomic status (23). Our results suggest that adolescent prevention and cessation programs should explicitly include young people with disabilities. Interventions should be designed in partnership with current or former smokers from the disability community and employ accommodations that support the participation of adolescents in program activities (24). For example, curriculum may need to be tailored and teaching modes adapted to accommodate students with visual, hearing, cognitive, or mobility impairments. Additionally, intervention materials and activities that include stories, images, and experiences of people with disabilities are more likely to be effective in reaching adolescents who feel marginalized because of their disability.

Both the American Academy of Pediatrics and the US Preventive Service Task Force recommend that primary care clinicians screen for and intervene on tobacco use in school-aged children and adolescents (25,26). Effective risk assessment requires that providers understand the susceptibility to and excess burden of tobacco use in adolescents with disabilities. Twenty-eight percent of our sample identified as having at least 1 disability, which is a substantial proportion. Yet, many clinicians receive little training in how to provide care to people with disabilities, and they may be vulnerable to focusing on the disability to such an extent that other health concerns are missed (27). Improving disability competency among clinicians, along with awareness of disability-related disparities, will better equip providers to have important tobacco use screening, prevention, and cessation conversations.

Finally, amid large increases in ENDS use among young people, a concerted and continued effort to educate parents, caregivers, educators, health care providers, and others about emerging nicotine delivery devices is essential. New products evolve quickly and keeping up with patterns of use among adolescents can be challenging. In January 2020, the US government banned the sale of flavored cartridge-based ENDS products that appeal to young people, including mint and fruit flavors (28). Almost as quickly as the ban took effect, disposable (ie, cartridge-less) fruit-flavored products flooded the high school market (29). Longitudinal studies suggest that adolescents who only use ENDS are nearly 3 times more likely to initiate smoking combustible products in the future compared with adolescents who do not use ENDS (4,30). Thus, these devices influence the uptake or continuation of other forms of tobacco. Novel public health efforts to minimize their use among all young people, with a focus on those at highest risk, are critical.
